# 1238. Evaluation of Children Exposed to Perinatal Hepatitis C in the US : A Literature Review

**DOI:** 10.1093/ofid/ofac492.1069

**Published:** 2022-12-15

**Authors:** Ezzeldin Saleh, Marcela Rodriguez, Subhash Chaudhary

**Affiliations:** Southern Illinois School of Medicine, Springfield, Illinois; Southern Illinois School of Medicine, Springfield, Illinois; Southern Illnois University School of Medicine, Springfield, Illinois

## Abstract

**Background:**

Incidence of Hepatitis C virus (HCV) infection is rising in the United States (US), largely due to the ongoing opioid epidemic. In children, perinatal transmission is the most common route of HCV infection, which is almost always asymptomatic. Therefore diagnosis is confirmed by HCV antibody testing by ≥18 months due to persistence of maternal antibodies. Identification of HCV-infected infants is critical as up to 80% develop chronic HCV infections with significant morbidity and mortality. In this review, we examine studies evaluating appropriate testing for perinatal HCV.

**Methods:**

We performed a systematic literature review with PubMed and Embase (through April 2022) for studies evaluating optimal testing of children exposed to perinatal HCV in the US. Full text articles in English were included and conference proceedings were excluded.

**Results:**

We analyzed 13 articles; all were published in the last decade with study periods spanning from 1993 to 2018 [Table1]. Most of the studies were retrospective, except for one prospective and two before-and-after intervention. Rates of optimal HCV testing showed vast regional differences and varied widely, with rates as low as 7%. Only 3 studies achieved ≥ 50% complete testing, with the highest rate (61%) achieved in a study that adopted EMR-reminders for testing. The two interventional studies showed over 40% increase of adequate testing. Interventions included early engagement of caregivers at time of birth with consultation, education, and close follow-up, EMR leverage to document HCV-exposure and send reminders for final testing at 18 months of age. Five studies retrospectively evaluated maternal and infant risk factors associated with perinatal HCV testing [Table2].

**Figure:**
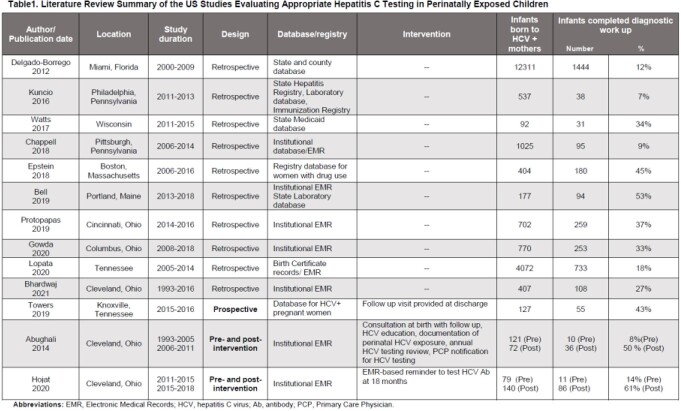

**Figure:**
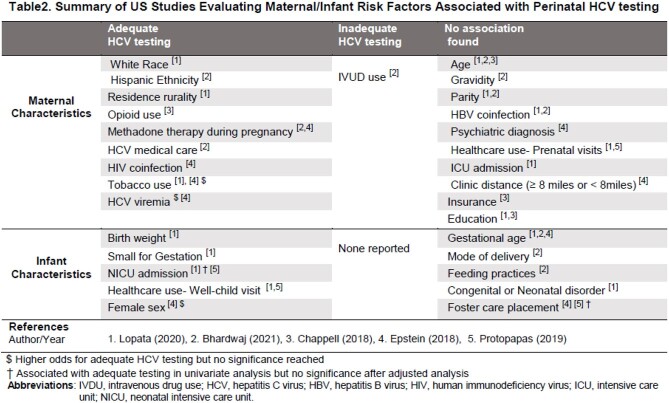

**Conclusion:**

Overall, rates of perinatal HCV appropriate testing were suboptimal and varied widely. Prospective and EMR-based interventional studies showed higher rates of testing. Innovative testing schemes, public health and social support programs, similar to perinatal HIV model, are strongly needed to substantially improve perinatal HCV management. Further prospective and interventional studies are needed to formulate effective guidelines for perinatal HCV evaluation, and to identify and address barriers and enablers to optimal perinatal HCV care.

**Disclosures:**

**All Authors**: No reported disclosures.

